# Do tragic movies about death make you think? Exploring effects of the role of death in the narrative structure of eudaimonic movies on viewers’ reflection, death attitudes, and posttraumatic growth

**DOI:** 10.1371/journal.pone.0323739

**Published:** 2025-05-19

**Authors:** Kobie van Krieken, Enny Das

**Affiliations:** Centre for Language Studies, Radboud University, Nijmegen, The Netherlands; Universitat Autònoma de Barcelona: Universitat Autonoma de Barcelona, SPAIN

## Abstract

Reflection has been found to play an important role in the effects of eudaimonic movies, but it is yet unclear which features of movies stimulate viewers’ reflection. This study tests whether the role of death in the narrative structure of movies about death affects viewers’ reflection and, subsequently, their attitudes towards death and posttraumatic growth. In a between-subjects experiment, participants watched two short movies in which death was either the *triggering event*, occurring at the beginning of the movies, or the *resolution*, occurring at the end of the movies. Prior to watching, viewers were reminded of their own death, the death of a loved one, or of eating breakfast (control condition). Their reflection, attitudes towards death, and posttraumatic growth were measured after watching. The results showed that for people who had been reminded of the death of a loved one, movies in which death was the *resolution* decreased reflection in the sense that viewers found the movies less thought provoking. No effects were found of narrative structure and mortality reminders on viewers’ death attitudes and posttraumatic growth. These results add to our understanding of how and when eudaimonic movies do and do not affect viewers’ reflection, death attitudes, and growth.

## Introduction

Eudaimonic entertainment is often described as meaningful entertainment that moves, touches or inspires the audience [1,2]. Eudaimonic films and series emphasize human virtues, such as care and courage, and typically display the threatening of fundamental human needs [3–5]. Watching such films and series has been linked to several positive outcomes. For example, eudaimonic entertainment can foster psychological well-being, social connectedness, as well as the ability to deal with difficulties [6,7].

Reflection plays an important role in the effects of eudaimonic entertainment. For example, viewers who were explicitly instructed to watch and reflect on a tragic movie (versus only watch) showed higher levels of reflection, which positively influenced their self-compassion [8]. In a similar study, participants were asked to watch a tragic movie or read the movie script and to write their reflections on the story afterwards [9]. Results showed that in both conditions, participants’ reflection on the tragic drama was associated with improvements in depressed mood and anxiety. A different study found that a more (versus less) moving film resulted in more reflective thoughts, which in turn resulted in a more positive experience of the film [10]. Similarly, a study in which participants watched a shortened version of the tragic movie *Atonement* showed that viewers who reported higher levels of sadness after watching the movie also displayed higher levels of reflection, which, in turn, increased their enjoyment of the movie. Moreover, reflective thoughts about close relationships were found to increase viewers’ life happiness [11]. Findings of these studies are in line with the contention that people turn to eudaimonic entertainment for specific motivations, such as the desire to search for meaning in life [1].

It is yet unclear, however, to what extent viewers’ reflection is stimulated by specific movie characteristics. The above studies either explicitly instructed viewers to reflect on the movie they watched [8,9] or exposed all participants to the same movie and used the level of viewers’ emotions to predict reflection [10,11]. Given the importance of reflection for the effects of eudaimonic entertainment, a relevant question is how, and under which conditions, eudaimonic movies might prompt *spontaneous* reflection in viewers. In this study we will therefore examine the role of narrative structure – a characteristic that has been found to influence entertainment experiences [12] – in viewers’ reflection. We will examine this role in the specific context of movies about death, for it has been argued and demonstrated that meaningful movies about death can help people to transcend their most fundamental fear, i.e., their fear of death [13,14]. Viewers’ reflection has been proposed as a potential explanatory mechanism for this effect [13], but this relation has yet to be tested.

It is furthermore not yet known if movies about death can also positively influence viewers’ attitudes towards life in terms of personal posttraumatic growth, although this can be expected based on studies showing that attitudes towards death are intimately connected to attitudes towards life [15,16]. Answering this question will reveal if reflecting on eudaimonic entertainment affects not only viewers’ self-compassion, mood, and happiness, as established in previous studies [8,9,11], but also their growth – which is considered a key component of psychological well-being [17]. The primary (a) and secondary (b) goals of this study are therefore to examine (a) if the role of death in the narrative structure of movies affects viewers’ spontaneous reflection, their attitudes towards death and their posttraumatic growth, and (b) if reflection explains for these effects.

In the following section we first discuss literature on the relation between eudaimonic entertainment and death attitudes, and explain why eudaimonic entertainment may also influence viewers’ attitudes towards life in terms of posttraumatic growth. We then elaborate on the narrative structure of stories, and movies in particular, and argue how the role of death in this structure might influence viewers’ reflection, attitudes, and growth.

### Eudaimonic entertainment and death attitudes

The link between eudaimonic movies and viewers’ attitudes towards death, in particular their fear of death, has been examined in several studies. These studies find their origin in central propositions of Terror Management Theory, which include that the human instinct of self-preservation combined with the human understanding that death is inevitable creates the potential for terror [18]. People therefore try to avoid death and thinking about death as much as possible. At the same time, and somewhat paradoxically, people are drawn to movies, series and books that often revolve around tragic events involving death, illness, loss and grief. As a solution to this paradox, it has been proposed that eudaimonic movies may “help people to cope with their own mortality by reminding viewers of the deeper meaning of life, or of the values and virtues that will persist even after their death” [19, p. 714].

In line with this reasoning, the recently developed *Terror Management Theory of Meaningful Entertainment* (TMT-ME) proposes that meaningful movies can be processed along two distinctive routes [13]. The first route holds that meaningful movies, in general, can function as an anxiety buffer against viewers’ fear of death, that is: viewers watch meaningful movies to suppress thoughts about death. The second route holds that when death is a central theme, meaningful movies can actually help people to overcome their fear of death, that is: “Movies about death can invite viewers to confront the fear of death at a safe aesthetic distance” and “invite them to embrace and transcend their fear of death” [13, p. 5]. The TMT-ME delineates three pre-conditions for this second route to be taken. First, viewers have to be reminded of death before watching the movie such that they have a motive to use the movie as a way to overcome their fear of death. Second, death has to be central to the movie such that viewers have the opportunity to learn about death from the movie. And third, death has to be meaningful such that viewers can engage in self-transcending experiences, which might be necessary to experience fear-transcendence.

Experimental studies have confirmed that meaningful movies that confront viewers with death can affect their attitudes towards death. In line with TMT-ME, such effects occur under the condition that viewers are reminded of death before watching the movie, i.e., that mortality is salient [13]. For the manipulation of mortality salience, studies typically use tasks that remind participants of their own death (mortality salience of self; MSS) or of the death of a loved one (mortality salience of a loved one; MSLO). Both types of mortality reminders can influence the effects of eudaimonic movies on viewers’ death attitudes. For example, in a study participants were asked to reflect on the mortality of a loved one (MSLO) or on eating breakfast (control condition) before watching one of three versions of a movie in which the main character intends to take assisted suicide: in the first version he dies and leaves his girlfriend with negative emotions, in the second version he dies and leaves his girlfriend with mixed emotions, and in the third version he chooses to live, a decision sparking positive emotions in his girlfriend [20]. Results showed that older (but not younger) reported lower levels of death avoidance after watching the ending with mixed emotions (compared to the two other endings), but only if they had been reminded about the death of a loved one. A different study demonstrated that people showed higher death acceptance after watching a scene with (versus without) a death portrayal, but only if they had been reminded of their own death (MSS) prior to watching [13].

Results of these previous studies indicate that meaningful movies about death may help viewers to actually approach rather than suppress death, specifically if they have been reminded about their own mortality or the mortality of a loved one. Although both MSS and MSLO can play a role in the effects of eudaimonic entertainment on viewers’ death attitudes, explicit comparisons between the two types of mortality reminders are necessary to fully understand their respective roles [20]. The current study will therefore make a direct comparison between MSS and MSLO reminders.

### Death attitudes and posttraumatic growth

The current study introduces the novel proposition that movies about death may not only have a positive impact on viewers’ attitudes towards death, but also on their attitudes towards life. The possibility of such an effect is substantiated, first, by studies demonstrating a strong link between death attitudes and life attitudes [15,21] and, second, by studies demonstrating positive effects of tragic movies on viewers’ well-being [8,11]. In the specific context of movies about death, these positive effects might lie in a renewed appreciation of life rather than, for example, increased levels of vitality [6]. Specifically, the effects of eudaimonic movies about death on viewers’ life attitudes can be expected to manifest as posttraumatic growth. Posttraumatic growth occurs when people perceive benefits emerging from traumatic experiences, such as life threatening illnesses, abuse, and bereavement [22]. Common benefits are perceived changes in the self, changed relationships with others, and a changed philosophy of life [22].

Movies often depict characters displaying posttraumatic growth: they struggle with difficulties, such as the death of a loved one, and ultimately overcome these difficulties and become better and stronger persons [cf. 23]. Through embodiment, spectators mentally and emotionally simulate the events in a story and the experiences of characters [24–26]. These simulations involve “embodied perceptions of what happens in a fictional world, as well as in the imagination constructing and participating in events” [27, p.11] and enable “immersive relationships with the narrated characters” [25, p.197]. Hence, viewers of eudaimonic movies can be expected to not only *watch* characters experiencing post-traumatic growth, but to also *simulate* this growth. Simulating a character’s post-traumatic growth may ultimately translate into actual personal growth. This might be particularly the case for people with a salient fear of death for it has been found that simulation is triggered by mortality reminders. Specifically, reminding people about the death of a loved one increases their engagement with tragic movies [28]. Viewers’ engagement with eudaimonic movies, and in particular their reflection on such movies, can furthermore be expected to be influenced by the role of death in the narrative structure of movies, as will be explained below.

### Death and the narrative structure of movies

Like any other type of narrative, movies consist of several recurring structural elements. The ordering of these elements, hence the narrative structure, has been found to influence the audience’s cognitive and emotional responses to the narrative [29,30]. Previous research has proposed a variety of classifications of the recurring elements in narratives [31–33]. For example, the structure of movies is often divided into three acts: a beginning offering the set-up of the narrative, a middle depicting a confrontation, and an ending revealing the narrative’s resolution [31].

The present study adopts the classification proposed by Labov and Waletzky [34], which has its origins in analyses of oral interactive narratives but can be applied to short audiovisual narratives as well [35], which are the focus of the current study. In this classification, narratives start with an *orientation* in which the spatiotemporal setting and characters are introduced. Then a *triggering event* occurs [also referred to as *inciting incident*; 36], which is the first, crucial event in the causal chain of events (*complicating actions*) which moves the narrative plot forward. This causal chain results in a *critical event*, the central event and often turning point or climax [36] of the story. Finally, the *resolution* refers to the event or events happening after the critical event, which depict the story’s outcome [see also 36].

Death often plays an important role in eudaimonic movies, and a character’s death can occur at different points in a movie. Hagin [37] differentiates between story-initial, story-intermediary, and story-terminating deaths. A story-initial death forms the beginning of a storyline and causes the subsequent events, whereas a story-terminating death is an effect of previous events and ends a storyline. Story-intermediary deaths occur in the middle, and are both an effect of previous events and a cause of subsequent events. In this study we distinguish story-initial deaths, specifically movies in which death is the *triggering event* of the narrative, from story-terminating deaths, specifically movies in which death is the *resolution* of the narrative.

Movies in which death is the triggering event – and, hence, forms a beginning – can be expected to stimulate viewers to reflect on the meaning of death for the characters involved. For example, the movie *Pieces of a Woman* (2020) starts with the death of main character Martha’s newborn baby. The remainder of the movie shows Martha’s grieving process, which causes the end of her marriage, but ultimately she seems to find peace with the loss of her baby and reconciles with her estranged mother. Movies like this, in which death is the triggering event, often relate death to a theme of growth [38] and they might invite viewers to reflect on death and how death, however devastating, may also spark positive changes and transformations in people.

Movies in which death is the resolution offer less room for reflection because in such movies death marks an ending rather than the beginning of a transformation; in these movies, death is typically related to a theme of separation [38]. For example, in the movie *I, Daniel Blake* (2016), main character Daniel becomes friends with Katie and her two children. At the end of the movie, Daniel dies from a heart attack. Movies like this show to a lesser extent how death can be meaningful compared to movies in which death is the triggering event. Movies of the latter type might therefore result in higher levels of reflection in viewers, which might in turn influence their attitudes towards death and life.

## Hypotheses

In sum, previous research has shown that eudaimonic films can positively influence viewers’ well-being [e.g., 6], and that reflection plays an important role in these effects [e.g., 8]. It has also been theorized and demonstrated that eudaimonic films about death can positively influence viewers’ attitudes towards death, provided that they have been reminded about death prior to watching [13,20]. Open questions are 1) to what extent reflection plays a role in these latter effects, 2) if films about death can also positively affect viewers’ attitudes towards life in terms of post-traumatic growth, 3) and if specific movie characteristics affect viewers’ spontaneous reflection. The current study aims to answer these questions by testing the following set of hypotheses. Combining theoretical frameworks about the structure of narratives and the role of death in the narrative structure of films [34,37] with the finding that the narrative processing of eudaimonic movies is triggered by mortality reminders [28], we hypothesize that:

H1: Compared to people who watch a eudaimonic movie in which death functions as a *resolution*, people who watch a eudaimonic movie in which death functions as a *triggering event* will show higher levels of reflection (H1a), in particular after being reminded of death (vs. control) prior to watching the movie (H1b).

Viewers mentally and emotionally simulate the journey of movie characters [24] who often display strength and growth as they face and overcome obstacles [23], which in particular is the case in movies in which death is the *triggering event*. Such movies might therefore lead to more positive attitudes towards death and life:

H2: Compared to people who watch a eudaimonic movie in which death functions as a *resolution*, people who watch a eudaimonic movie in which death functions as a *triggering event* will show more positive attitudes towards death in general (H2a), in particular after being reminded of death (vs. control) prior to watching the movie (H2b).

H3: Compared to people who watch a eudaimonic movie in which death functions as a *resolution*, people who watch a eudaimonic movie in which death functions as a *triggering event* will experience more post-traumatic growth (H3a), in particular after being reminded of death (vs. control) prior to watching the movie (H3b).

Finally, because reflection has been found to mediate the impact of eudaimonic movies on various aspects of viewers’ well-being [8,9,11], we hypothesize that:

H4: Reflections on the movie’s death mediate the impact (moderated by mortality salience) of movie type on posttraumatic growth and death attitudes.

**Fig 1:**
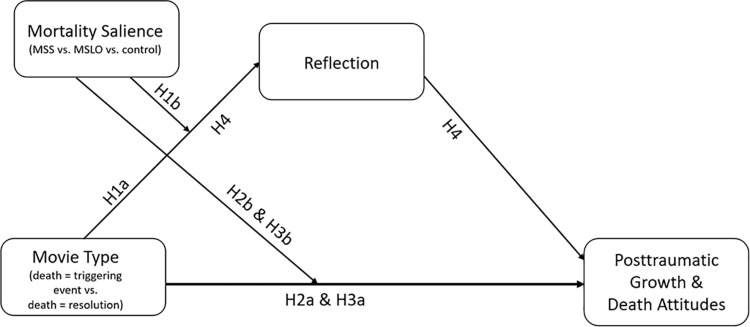
Conceptual model of the hypothesized relations between narrative structure, mortality salience, reflection, posttraumatic growth and death attitudes.

The hypotheses are visualized in the conceptual model presented below in Fig 1.

### Pilot study

A pilot study was conducted to select the experimental stimuli for the main study. The study was approved by the Ethics Assessment Committee Humanities of Radboud University (ETC-GW number 2020-8854). The data for the pilot study were collected between September 21 and September 29, 2021.

We conducted an extensive online search for movies about death and loss that could be considered as eudaimonic based on descriptions of eudaimonic entertainment in previous studies: the movies had to portray a crucial life event [14] that threatens a fundamental human need [5] and the movies had to show human vulnerability [39]. The search resulted in a set of eight short movies that differed with respect to the role of death in the narrative structure of the movie. In four movies, death was the *resolution* of the story: *The Song of the Rain*, *Dad, Message from Tar Creek* and *North*. For example, *The Song of the Rain* shows a love story of a woman and a man, which, at the end of the movie, comes to an end when the man dies. In *Dad*, the main character is a teenage girl who is angry with and disappointed in her father, who cannot buy her all the things she wants. At the end of the movie he dies in a car accident, leaving his daughter guilt-ridden. Hence, in these as well as the two other *resolution* movies, death was the outcome of the story. In the other four movies, death was the *triggering event*: *Like Father, Grief, Street Flame,* and *Lost in the Smoke*. In the first scene of *Like Father*, a teenage boy is playing baseball with his father when his father is hit by a car. His death makes it hard for the boy to concentrate on his school work, but eventually he manages to step up and by the end of the movie his grades are better again. In *Street Flame*, a teenage girl dies in a car accident. Her friends find it hard to deal with her death, but along the road they seem to find some closure. Hence, in these as well as the two other *triggering event* movies, death was the beginning of the story and initiated the narrative plot.

There were considerable differences between the movies in terms of length, use of music and animations, and use of subtitles, among other. The main goal of the pilot study was to rule out the possibility that these differences could affect viewers’ engagement with and understanding of the movies, which could in turn influence the study’s results.

A total of 117 Dutch people participated in the pilot study (52% female; *M*_*age*_ = 29.3 (*SD* = 10.2)). They were recruited via Prolific and received a monetary compensation. After providing written consent, each participant watched two of the eight movies (both either in the *resolution* condition or in the *triggering event* condition). After each movie they completed a questionnaire. In line with previous research [e.g., 40], this study conceptualizes narrative engagement as a multidimensional experience. Four dimensions of participants’ engagement with the narrative were measured. Following previous studies on eudaimonic entertainment [4,13], we measured viewers’ experiences of being moved, being absorbed, and their identification with the characters. First, their experience of ‘being moved’ by the movie was measured with nine items derived from previous work [4,41], such as “I was touched by the movie” and “I felt emotional while watching the movie” (α =.94). This measure was included to assess eudaimonic experiences. Second, their experience of being absorbed by the movie was measured with four items [13], such as “I was fully focused on the movie while watching” and “While watching the movie, it seemed as if I was there in my thoughts” (α =.90). Third, their identification with the narrative characters was measured with three items [13], such as “While watching I imagined what it would be like to be in the characters’ position” and “While watching I imagined what it must have been like for the characters to experience the events” (α = .90). In addition, viewers’ understanding of the movie was included as a fourth dimension of narrative engagement [see 40] so that we were able to select movies that were equally understandable. This dimension was measured with 3 items [40], such as “I had a hard time recognizing the thread of the story” and “My understanding of the characters is unclear” (α = .88). All responses were provided on a 7-point Likert-scale (1 = totally disagree, 7 = totally agree).

To assess the extent to which the various movies focused on death, participants were asked to select three themes which they considered to be the movie’s main themes out of a list of fourteen themes, including “Death and loss” but also “Family”, “Love”, “Nature” and “Politics”, among other. Participants were furthermore asked to respond to the statements “I feel that death was a major theme in this movie” and “I think that death was an important theme in this movie” (r = .84**; 1 = totally disagree, 7 = totally agree).

We also assessed if the movies differed in the expression of attitudes towards death. Wong et al. [42] distinguish five death attitudes: Fear of Death (reflecting one’s anxiety of death and dying), Death Avoidance (reflecting one’s tendency to avoid death and death thoughts as much as possible), and three types of acceptance: Approach Acceptance (reflecting one’s belief in a better existence in an afterlife), Escape Acceptance (reflecting one’s view that death can be a relief), and Neutral Acceptance (reflecting one’s recognition that death is a natural part of life). We measured the extent to which viewers thought that the movies/characters expressed these attitudes. For each attitude, one item was included: “This movie shows that death is an entrance to a place of ultimate satisfaction” (Approach Acceptance); “The characters in this movie have an intense fear of death” (Fear of Death); “In this movie the characters try to have nothing to do with the subject of death” (Death Avoidance); “In this movie death is considered a liberation” (Escape Acceptance); “This movie shows that death is a natural aspect of life” (Neutral Acceptance) (1 = totally disagree, 7 = totally agree).

Participants’ attitude towards the movie was measured with four items on a seven point semantic scale, including: “I found the movie boring – fascinating” and “I found the movie unattractive – attractive” (α = .91).

Finally, two items were included to check if participants were sensitive to the difference between the two movie types in terms of the role of death in the narrative structure: “In this movie death mainly played a role in the beginning of the story” and “In this movie death mainly played a role in the end of the story”. Responses were provided on a 7-point Likert-scale (1 = totally disagree, 7 = totally agree).

## Results

The results were analyzed with IBM SPSS Statistics 27. First, a series of MANOVA’s, ANOVA’s and chi-squared tests was run to compare each of the four *resolution* movies individually to the four *triggering event* movies. Movies which differed significantly from one another on one or more of the dependent variables were considered inappropriate comparison materials for the main study. For example, participants were more strongly moved by the movie *Dad (resolution)* compared to *Street Flame (triggering event)*, but they felt that death was thematized to a greater extent in *Street Flame* compared to *Dad*.

Results of these initial analyses indicated that both *Song of the Rain (resolution)* and *Message from Tar Creek (resolution)* were comparable to both *Street Flame (triggering event)* and *Lost in the Smoke (triggering event)*. To confirm this impression, additional analyses were performed in which these two *resolution* movies were compared to these two *triggering event* movies. We aimed for two movies per condition in the main study to rule out movie-specific effects. The results are shown in Table 1.

**Table 1:** Means and standard deviations for narrative engagement, attitude, thematization of death, death attitudes, and the manipulation check in resolution movies vs. triggering event movies.

	Death as Resolution (Song of the Rain + Message from Tar Creek)	Death as Triggering Event (Street Flame + Lost in the Smoke)
*Narrative engagement*		
Being moved	3.61 (1.30)	3.76 (1.18)
Absorption	4.63 (1.46)	4.76 (1.31)
Narrative understanding	4.25 (1.66)	4.54 (1.31)
Identification	4.23 (1.49)	4.89 (1.28)
*Death attitudes*		
Fear of death	2.49 (1.44)	2.67 (1.06)
Death avoidance	3.02 (1.74)	2.85 (1.38)
Approach acceptance	2.74 (1.43)	2.59 (1.28)
Escape acceptance	3.51 (2.01)	2.87 (1.49)
Neutral acceptance	4.09 (1.73)	3.67 (1.81)
*Attitude towards movie*	4.79 (1.23)	4.58 (1.10)
*Thematization of death*		
Death picked as main theme (%yes)	93%	87%
Thematization of death	5.38 (1.59)	5.36 (1.59)
*Manipulation check*		
Death plays role in beginning of movie	2.42 (1.58)^***^	3.94 (1.76)^***^
Death plays role in end of movie	5.75 (1.52)^***^	4.19 (1.68)^***^

Note: 1 = low, 7 = high;

*** p < .001.

A MANOVA revealed no significant effect of movie type on engagement with the movie (*F*(4, 106) = 2.40, *p* = .057, partial η^2^ = .082). Similarly, a MANOVA showed that there was no effect of movie type on the degree to which various death attitudes were expressed in the movie (*F*(5, 105) = 1.16, *p* = .336, partial η^2^ = .052). A one-way ANOVA showed that there was neither a significant effect of movie type on attitude towards the movie (*F* < 1, partial η^2^ = .007). A chi-squared test revealed no significant relation between movie type and the percentage of participants who picked death as one of the main movie themes (χ^2^(1) = 1.10, *p* =  .295, Cramer’s *V* = .099). There was also no effect of movie type on perceived thematization of death in the movie (*F* < 1, partial η^2^ = .000). Together, these results indicate that the two resolution movies and the two triggering event movies were comparable in terms of viewers’ engagement and attitude as well as the thematization of death and expression of death attitudes.

For the manipulation check, a MANOVA revealed a significant effect of condition on the perceived role of death in the movie (*F*(2, 108) = 23.24, *p* < .001, partial η^2^ = .301). Subsequent univariate analyses showed that participants who watched a movie in which death was the triggering event, more strongly found that death mostly played a role in the beginning of the movie compared to participants who watched a movie in which death was the resolution (*F*(1, 109) = 23.02, *p* < .001, partial η^2 ^= .174). Likewise, participants who watched a movie in which death was the resolution, more strongly found that death mostly played a part in the end of the movie compared to participants who watched a movie in which death was the triggering event (*F* 1, 109) = 26.70, *p* < .001, partial η^2 ^= .197). These results indicate that the role of death in the narrative structure differed as anticipated between the two movie conditions. Combined with the lack of differences between the movie conditions in terms of viewers’ engagement and attitudes and the thematization of death and expression of death attitudes in the movies, these results support the selection of these four movies as stimuli for the main study.

## Main study

### Design and procedure

An online study was designed with a 3 (Mortality Salience: Self vs. Loved One vs. Control) X 2 (Movie Type: Death as Triggering Event vs. Resolution) between-subjects design. The study was approved by the Ethics Assessment Committee Humanities of Radboud University (ETC-GW number 2020–8854). Participants were recruited through Prolific between October 12, 2021 and October 23, 2021.

Upon starting the study, participants were directed to a Qualtrics survey with a cover page explaining the goal of the study, which was described as obtaining opinions about sad movies. Participants who agreed to participate provided written informed consent. Then they were randomly assigned to one of the three experimental conditions and, subsequently, they were randomly assigned to one of the four movies. They were asked to watch the full movie and were unable to move on to the questions until the movie had ended. Inspection of the open-ended question (see below) confirmed that the participants had watched the movie and were able to reflect on it.

After completing the study, participants received a monetary compensation for their time (£3,75). The mean duration of completing the study was 24 minutes. If it took participants longer than twice the standard deviation of the mean duration to complete the study, their responses were excluded from the dataset. This was done to ensure that participants’ responses to the stimuli were non-delayed. Data from two participants (who spent respectively 3 and 21 hours on the study) were deleted from the dataset for this reason.

### Mortality Salience manipulation

*Mortality Salience of Self* was manipulated by asking participants (a) to briefly describe the emotions that the thought of their own death awakens in them and (b) to describe, as specifically as they could, what they think will happen to them as they physically die and once they are physically dead [43]. *Mortality Salience of a Loved One* was manipulated in the same way, but in this condition participants were asked to think of a loved one and (a) to briefly describe the emotions that the thought of the death of their loved one awakens in them and (b) to describe, as specifically as they could, what they think will happen to their loved one as this person physically dies and once this person is physically dead [28]. Participants were also asked to report who they were thinking of. In the control condition, participants were asked (a) to briefly describe the emotions that the thought of eating breakfast awakens in them and (b) to describe, as specifically as they could, what they think happens to them physically when they are eating breakfast [28]. We used two, rather than one specific mortality salience manipulation to enable a direct comparison between the two types of mortality reminders.

### Stimulus materials

The stimulus materials were the four movies which were selected based on the results of the pilot study. In the movies *Song of the Rain* and *Message from Tar Creek*, death functions as the resolution in the narrative structure, i.e., the outcome of the story. *Song of the Rain* is an 8-minute animated film depicting the love story of a young man and woman in China. The movie first shows how they fall in love, spend time together and start living together. Then the man is sent to war. An explosion occurs and he dies. *Message from Tar Creek* is a 4-minute film about a young couple in love heading out to nature by car. They enjoy the surroundings and each other’s company; and they kiss and swim. Near the end of the movie, they discuss the future and the boy – Dakota – says that he will leave soon. During the movie, a voicemail is played that is recorded almost 20 years later in time and that reveals that the two lost touch a long time ago. The girl – now a woman with a family of her own – is leaving a message for Dakota even though she recently learned he is dead; it is suggested that he committed suicide.

In the two other movies, *Street Flame* and *Lost in the Smoke*, death functions as the triggering event in the narrative structure, i.e., a crucial event triggering a chain of events towards the climax of the story. *Street Flame* is an 11-minute movie about a young skate crew. In the beginning of the movie, one of the crew members, Jinx, dies in an accident. Her death leaves her friends struggling with questions and their emotions. They try to find closure while continuing their skating adventures. When Jinx’s mother gives Jinx’s skateboard to her friends, they finally find a way to commemorate Jinx and let go of the past: they start a bonfire and set Jinx’s skateboard on fire, a ritual to honor her life. By means of this ritual, the friends find a way to move on and to strengthen their friendship. This act thus symbolizes their posttraumatic growth. *Lost in the Smoke* is a 10-minute movie about Caleb, a teenager who loses his parents in a fire. Struggling with his parents’ death, he starts getting into trouble. A local police officer suggests Caleb should aim for a football career, but he keeps getting into trouble. One day he and his friends set out to reclaim a football that was stolen from Caleb, taking a knife with them. The police intervene and arrest Caleb’s friends. Caleb runs away but the local police officer catches up with him. However, she does not arrest him but says that they will see what they can do about his football, and she lets him go. The final scenes show how Caleb is playing football again instead of getting into trouble, which symbolizes his posttraumatic growth: he has become a wiser person.

Summarizing, in all four movies the main characters are confronted with the death of a loved one. All four deaths are similar in the sense that these are all tragic, “non-deserved” deaths of “good” characters that were loved by the main characters. The only difference between the conditions was that in the two movies in the *triggering event* condition, this death triggers a chain of events depicting the struggles and eventual growth of the bereaved, whereas in the two movies in the *resolution* condition, death marks the ending of the story. This does not imply, however, that the resolution was outspoken negative in the latter condition and outspoken positive in the former. Rather, the resolution of all four movies can be qualified as “neutral”: the resolutions do not show explicit signs of either negativity or positivity.

### Measures

*Posttraumatic growth* was measured with the Short form of the Posttraumatic Growth Inventory [44; α = .92]. This scale measures five components of posttraumatic growth, each with two items: growth in terms of (1) Relating to others (e.g., “I have a greater sense of closeness with others”, (2) New Possibilities (e.g., “I am able to do better things with my life”), (3) Personal Strength (e.g., “I discovered that I’m stronger than I thought I was”), (4) Spiritual Change (e.g., “I have a better understanding of spiritual matters”), and (5) Appreciation of Life (e.g., “I have a greater appreciation for the value of my own life”). Responses were given on a 7-point Likert scale (1 = fully disagree; 7 = fully agree). The scale is generally used to produce a single overall indicator of posttraumatic growth rather than five separate indicators corresponding to the five dimensions [44], which is why we choose to treat it as a unidimensional variable in the current study.

*Reflection* was measured in two ways. First, an open thought listing procedure was used to assess participants’ reflection on the movie. Participants were asked to think about the movie they had seen and to write down the thoughts they had while watching the movie. This instruction was followed by 15 blank text boxes. Participants were asked to use a separate text box for each thought they wanted to write down. Each reported thought was manually coded on two variables. First, it was determined if the thought was a reflective thought or not. A thought was considered to be reflective if it established a connection between the movie on the one hand and the viewer’s personal experience, moral judgments, or the real world in general on the other. Second, it was determined if the thought was related to death. A thought was qualified as a death-related thought if it included one or more words referring to death, loss, grief, bereavement, funeral, memorial, heaven, life after death, or finding closure.

As a second measure of reflection, two subscales of the Appreciation scale [45] were used: the Thought-Provoking subscale (α = .90), consisting of three items (e.g., “The movie was thought provoking”), and the Lasting Impression subscale (α = .93), also consisting of three items (e.g., “This movie will stick with me for a long time”). The Thought-Provoking scale was used as an indication of the degree to which the movies led viewers into a reflective state, and the Lasting Impression scale was used as an indication of viewers’ prospective reflection. Responses were given on a 7-point Likert scale (1 = fully disagree; 7 = fully agree).

*Attitudes towards death* were measured with the 15-item Death Anxiety Scale [46; α = .84]. Example items are “I am very much afraid to die”, “The thought of death never bothers me”, and “The subject of life after death troubles me greatly”. Responses were given on a 7-point Likert scale (1 = fully disagree; 7 = fully agree). Because previous research has shown that this scale taps into different dimensions of peoples’ death attitudes [e.g., 47,48], a factor analysis was conducted. A Principal Components Analysis with direct oblimin rotation showed a 4-factor solution explaining 58.26% of the variance. Two items crossloaded on two factors. The item with the highest loadings (“The sight of a dead body is horrifying to me”) was deleted, after which the analysis was run again. This resulted in a 3-factor solution explaining 53.40% of the variance. The first factor consisted of 7 items about pain (“I fear dying a painful death”), illness (“I am really scared of having a heart attack), war (“I shudder when I hear people talking about a World War III”), and time (“I am often distressed by the way time flies so very rapidly”). This dimension was therefore labeled as *Fear of Pain, Illness, War and Shortness of Life* (α = .75). The second dimension consisted of 5 items about death and dying (“I am very much afraid to die”; “The subject of life after death troubles me greatly”). This dimension was labeled *Fear of Death and Dying* (α = .83). The third dimension consisted of two items: “The thought of death seldom enters my mind” (R) and “I feel that the future holds nothing for me to fear (R)”. This dimension was labeled *Death Thoughts and Fear of Future* (r = .38**).

At the end of the survey, participants were asked to report their first language, age, gender, and level of education. They were also asked if they had ever lost a loved one, whose death still had an impact on their life. If they answered this question with “yes”, they were subsequently asked to indicate how much this loss still affected them (1 = not so much; 7 = a lot).

### Participants

A total of 429 native speakers of Dutch started the study, of whom 372 successfully completed the study. Their age ranged from 21 to 75 (*M* = 33.6, *SD* = 10.4). About half (50.8%) of the participants was female; 48.1% was male and 1.1% indicated having a different gender. Education level varied from university (49.5%) to higher professional/applied science (32.8%) and intermediate vocational training (7.8%). An additional 8.3% of the participants selected high school as education level, 0.5% primary school, and 1.1% selected ‘other’. A majority (62.9%) of the participants indicated having lost a loved one whose death still had an impact on their daily life. The mean impact of this loss was 4.57 (*SD* = 1.42) on a 7-point scale. Participants were distributed equally across experimental conditions in terms of their age (*F* < 1), gender (χ^2^(10) = 3.27, *p* = .974), education level (χ^2^(25) = 33.80, *p* = .112), their reported loss of a loved one (χ^2^(5) = 3.80, *p* = .578) and the impact of that loss (*F* < 1).

### Statistical analyses

The statistical analyses consisted of three parts. First, correlation analyses were performed to explore the relations between the various mediator variables (reflection) and dependent variables (death attitudes and posttraumatic growth). Second, analyses of variance were performed to examine the main and interaction effects of the role of death in the narrative structure and mortality salience on the mediating and dependent variables, testing Hypotheses 1–3. Third, to test Hypothesis 4, moderated mediation analyses were performed using PROCESS model 8 (REF). Age and participants’ experience with the loss of a loved one were included as covariates in all analyses. The results were analyzed with IBM SPSS Statistics 27 and the PROCESS macro [49].

## Results

### Preliminary analyses

Correlation analyses were performed to assess the relationships between the intermediate reflection variables and the dependent variables. Reflection in terms of viewers’ perception of the movie’s Lasting Impression was positively related to viewers’ posttraumatic growth (.62**) and their Fear of Pain, Illness, War and Shortness of Life (.21**). Reflection in terms of viewers’ perception of the degree to which the movie was Thought Provoking was positively related to their posttraumatic growth (.66**), their Fear of Pain, Illness, War and Shortness of Life (.21**) and their Fear of Death and Dying (.14**). Reflection in terms of viewers death-related thoughts was positively related to their posttraumatic growth (.15**), their Fear of Pain, Illness, War and Shortness of Life (.16**) and their Fear of Death and Dying (.14**). Finally, reflection in terms of viewers’ reflective thoughts was positively related to their posttraumatic growth (.17**).

### Main analyses

Table 2 provides an overview of the means and standard deviations for all variables as a function of movie condition and mortality salience.

**Table 2:** Means and standard deviations for the intermediate and dependent variables as a function of movie condition and mortality salience.

	Death as Resolution				Death as Triggering Event			
	Control	MSS	MSLO	Total	Control	MSS	MSLO	Total
Death-related thoughts (*n*)	1.48 (2.39)	1.11 (0.99)	0.75 (0.85)	1.11 (1.58)	0.77 (1.12)	0.96 (1.30)	0.93 (1.13)	0.89 (1.18)
Reflective thoughts (*n*)	1.06 (1.92)	0.82 (1.32)	0.86 (1.33)	0.91 (1.54)	0.60 (1.11)	1.05 (1.67)	1.13 (1.99)	0.92 (1.63)
Lasting Impression	2.92 (1.37)	2.99 (1.38)	2.44 (1.45)	2.78 (1.42)	2.77 (1.60)	2.47 (1.51)	2.89 (1.53)	2.72 (1.55)
Thought Provoking	4.45 (1.43)	4.46 (1.48)	3.76 (1.75)	4.22 (1.59)	4.32 (1.77)	3.92 (1.66)	4.32 (1.61)	4.19 (1.68)
Posttraumatic Growth	3.35 (1.21)	3.37 (1.15)	3.06 (1.10)	3.26 (1.16)	3.33 (1.23)	3.05 (1.31)	3.25 (1.22)	3.21 (1.25)
Fear of Pain, Illness, War and Short-ness of Life (Death Attitudes dimension 1)	4.10 (0.96)	4.34 (1.10)	3.88 (1.10)	4.11 (1.07)	4.02 (1.26)	4.16 (1.12)	3.87 (1.13)	4.01 (1.18)
Fear of Death and Dying (Death Attitudes dimension 2)	3.64 (1.36)	3.74 (1.42)	3.31 (1.17)	3.56 (1.33)	3.77 (1.37)	3.58 (1.38)	3.47 (1.46)	3.61 (1.40)
Death Thoughts and Fear of Future (Death Attitudes dimension 3)	4.65 (1.28)	4.45 (1.38)	4.38 (1.41)	4.49 (1.36)	4.48 (1.38)	4.44 (1.47)	4.48 (1.24)	4.46 (1.36)

Note: 1 = low, 7 = high. MSS = Mortality Salience of Self; MSLO = Mortality Salience of a Loved One.

Hypothesis 1 proposed that, compared to people who watched a movie in which death functions as a resolution, people who watched a eudaimonic movie in which death functions as a triggering event would show higher levels of reflection (H1a), in particular after being reminded of death (vs. control) prior to watching the movie (H1b). The results indicated that there was no main effect of movie condition on any of the four reflection measures (all *F*’s < 1, all partial η^2^’s = .000). H1a was therefore not supported. There were also no main effects of mortality salience on the reflection measures (Lasting Impression: *F* < 1, partial η^2^ = .003; Thought Provoking: *F*(2, 364) = 1.35, *p* = .260, partial η^2^ = .005; Death-related thoughts: *F*(2, 364) = 1.27, *p* = .282, partial η^2^ = .005; Reflective thoughts: *F* < 1, partial η^2^ = .009).

**Fig 2:**
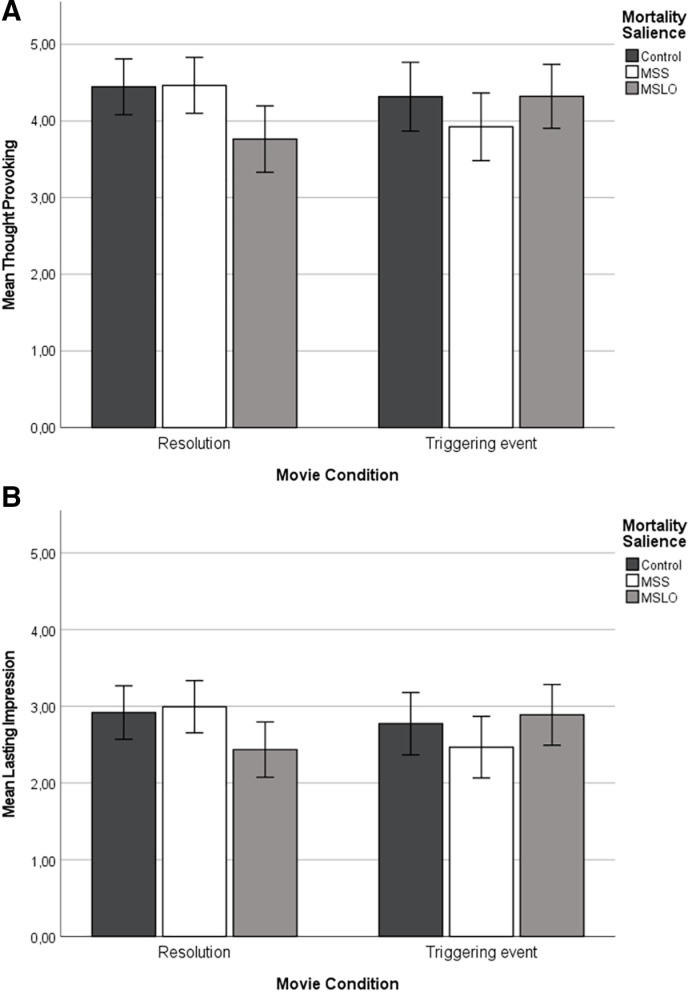
(a) Mean scores for the *Thought Provoking* measure per movie condition and mortality salience condition; (b) Mean scores for the *Lasting Impression* measure per movie condition and mortality salience condition.

For the Thought Provoking measure, a significant interaction effect was found (*F*(2, 364) = 3.89, *p* = .021, partial η^2^ = .006). Post hoc analyses (Bonferroni) showed that in Resolution movies, MSS led to more reflection than MSLO (*p* = .033) (see Fig 2a). The Control condition also led to more reflection than MSLO (*p* = .045). There was no difference between MSS and Control (*p* = 1.00). In Triggering event movies, there were no significant differences between the mortality salience conditions (*p*’s > .608). These findings do not support H1b.

There was also an interaction effect between movie condition and mortality salience for the Lasting Impression measure (*F*(2, 364) = 3.86, *p* = .022, partial η^2^ = .011). However, post hoc analyses were unable to probe the interaction (see Fig 2b for the mean scores per conditions).

Finally, there were no interaction effects between movie condition and mortality salience on the number of viewers’ Death-related thoughts (*F*(2, 364) = 1.03, *p* = .357, partial η^2^ = .017) nor on their Reflective thoughts (*F*(2, 364) = 1.98, *p* = .140, partial η^2^ = .011). H1b is not supported.

Hypothesis 2 proposed that compared to people who watched a movie in which death functions as a resolution, people who watched a movie in which death functions as a triggering event would experience more positive attitudes towards death (H2a), in particular after being reminded of death (vs. control) prior to watching the movie (H2b). A MANOVA was run to test the effects of movie condition and the interaction effects between movie condition and mortality salience, on the three death attitudes dimensions. Results showed no effect of movie condition on death attitudes (*F* < 1, partial η^2^ = .004). There was also no main effect of mortality salience on death attitudes (*F* < 1, partial η^2^ = .011), nor was there an interaction effect between movie condition and mortality salience (*F* < 1, partial η^2^ = .015). Hypothesis 2a and 2b were therefore not supported.

Finally, hypothesis 3 predicted that compared to people who watched a movie in which death functions as a resolution, people who watched a eudaimonic movie in which death functions as a triggering event would experience more posttraumatic growth (H3a), in particular after being reminded of death (vs. control) prior to watching the movie (H3b). An ANOVA showed no effect of movie condition on posttraumatic growth (*F* < 1, partial η^2^ = .009), no effect of mortality salience on posttraumatic growth (*F* < 1, partial η^2^ = .014), and neither an interaction effect between movie condition and mortality salience (*F* (2, 366) = 1.41, *p* = .246, partial η^2^ = .007). Hypothesis 3a and 3b were therefore not supported.

The fourth and final hypothesis proposed that the joint (interaction) effect of movie condition and mortality salience on posttraumatic growth and death attitudes would be mediated by viewers’ reflection. To test this hypothesis, we first ran an indirect effects model (PROCESS Model 8 [49]) with movie condition as independent variable, posttraumatic growth as dependent variable, the Thought-provoking reflection measure as mediator, and with mortality salience as moderator of the relationship between movie condition and the Thought-provoking reflection measure posttraumatic growth as well as the relationship between movie condition and posttraumatic growth. Age and personal experience with loss were included as covariates. The index for moderated mediation was not significant (MSS Dummy: -.22; CI -.60,.15; MSLO Dummy:.31, CI -.07, 71).

Next, we ran this model with the first dimension of death attitudes (Fear of Pain, Illness, War, and Shortness of Life) as dependent variable. Again, the index for moderated mediation was not significant (MSS Dummy: -.06; CI -.18,.04; MSLO Dummy:.08; CI -.02,.21). The model with the second dimension of death attitudes (Fear of Death and Dying) did not yield a significant index for moderated mediation either (MSS Dummy: -.05; CI -.17,.03; MSLO Dummy:.07; CI -.01,.22). Finally, the model with the third dimension of death attitudes (Death Thoughts and Fear of Future) also did not yield a significant moderated mediation index (MSS Dummy:.01; CI -.05,.08; MSLO Dummy: -.01; CI -.08,.07). These results do not provide support for H4.

## Conclusion and discussion

The primary goal of this study was to examine the role of death in the narrative structure in the effects of eudaimonic movies on viewers’ reflection, death attitudes, and posttraumatic growth. It was expected that movies in which death is a triggering event (versus a resolution) would increase viewers’ reflection, in particular when death is salient. The results were not in line with these expectations. For most of the reflection measures, no effect of movie type was found. For one reflection measure, the degree to which viewers thought the movie was thought provoking, the opposite effect was found: for movies in which death was the resolution (rather than the triggering event), mortality salience of self resulted in more reflection than mortality salience of a loved one. If people were reminded of their own mortality, a movie ending with the death of a character thus stimulated reflection such that viewers find the movie more thought provoking.

An explanation for this unexpected finding could be that movies in which death is the resolution have a more impactful end than movies in which death is the triggering event, which could in turn have led to higher levels of thought provocation. Note, however, that the pilot study showed that the two types of movies were equally absorbing and moving, which makes this explanation somewhat less likely. It is for this reason also unlikely that this effect could be explained as a recency effect, i.e., that reflection was higher for the movies in which death was the resolution because for these movies people remembered the death scene more clearly since it came last. It is furthermore important to keep in mind that the effect of resolution movies on thought provocation was not found for viewers who were reminded of the mortality of a loved one. Moreover, the control condition also led to more thought provocation than the condition in which viewers were reminded of the mortality of a loved one. This means that mortality does not necessarily have to be salient for the role of death in the narrative structure to have an effect on thought provocation. TMT-ME [13] proposes that mortality salience is a necessary condition for meaningful movies to influence viewers’ death attitudes, Initial studies support this proposition, but it remains unclear how these effects come about [13]. Results from the current study add to these studies by showing that mortality does not necessarily have to be salient for the occurrence of effects on viewers’ reflection. Future research should further test the full causal path from mortality salience elicitation to the processing of meaningful movies about death to effects on viewers’ death attitudes.

The results furthermore showed that the role of death in the narrative structure did not affect viewers’ death attitudes and posttraumatic growth, neither directly nor indirectly via reflection. Whereas the study by Das and De Graaf [13] showed that the type of ending (positive, negative, mixed) plays a role in the effects of eudaimonic movies on viewers’ death attitudes, the role of death in the narrative structure does not appear to be a relevant characteristic. Likewise, whereas the study by Das and Te Hennepe [28] showed that mortality salience of a loved one (but not of self) increases mixed affect for eudaimonic movies with a meaningful (but not with an open) ending, the current study showed that mortality salience of self (but not of a loved one) results in stronger thought provocation for eudaimonic movies in which death is the resolution (but not the triggering event). These diverging findings might point towards complex interactions between movie characteristics, the nature of mortality salience, and specific aspects of viewers’ processing, and stress the importance of direct comparisons between mortality salience of self and mortality salience of a loved one in testing the impact of meaningful movies on viewers’ fear of death. Future research is necessary to examine these interactions in more detail, and to further delineate the conditions under which eudaimonic movies affect certain aspects of viewers’ processing, and with which specific consequences.

### Reflection, death, and posttraumatic growth

The secondary aim of this study was to test whether reflection would explain potential effects of the role of death in the narrative structure of movies on viewers’ death attitudes and posttraumatic growth. Although the results of this study did not find any evidence for an indirect effect of the role of death in the narrative structure of eudaimonic movies on viewers’ death attitudes and posttraumatic growth through reflection, several findings are worthwhile to further discuss. Specifically, we observed a consistent pattern of positive correlations between the reflection measures on the one hand and the various dimensions of death anxiety and posttraumatic growth on the other hand. Death anxiety was also positively correlated with posttraumatic growth. These findings give rise to several considerations and questions.

First, the positive relation between reflection and posttraumatic growth is in line with previous studies which showed positive relations between reflection and self-compassion [8], reflection and improvements in depressed mood and anxiety [9], and reflection and life happiness [11]. Whereas these combined findings point towards a general relation between reflection on tragic movies and well-being, this appears to be different for viewers’ death anxiety, which was found to increase rather than decrease as their reflection increases. This could imply that for certain well-being aspects, such as death anxiety, conscious reflection on tragic movies might actually be disadvantageous rather than beneficial. Future research could test this possibility by measuring a range of well-being aspects, including those used in studies finding positive effects of reflection [8,9,11], and aspects related to existential fears such as death anxiety.

Alternatively, it could also be the case that an increase in death anxiety is actually beneficial in the sense that it can lead to growth. Our findings showed that people who were more afraid of death scored higher on posttraumatic growth. This could imply that an increase in death anxiety is a requirement for posttraumatic growth; that is, people might need a trauma before posttraumatic growth can be experienced. Because our findings are correlational, future studies are necessary to test the possibility of a causal relation between death anxiety and posttraumatic growth. Such studies could also address the discrepancy between our finding that death anxiety is positively related to posttraumatic growth and findings from previous studies which showed that people who feel that their life is meaningful (which is one of the aspects of posttraumatic growth) are less afraid of death [15,16,50].

Second, the overall levels of reflection were rather low, with mean scores hovering below or slightly above the neutral midpoint of the scales. Participants furthermore reported on average one death-related thought and one reflective thought. Results from previous studies show a similar picture: reflection scores were below or around the neutral midpoint of the scales in the studies by Knobloch et al. [11] and Bartsch et al. [10], and participants in the latter study reported on average less than two thoughts. The question is, then, to what extent people truly and spontaneously reflect on meaningful films. Perhaps people do not readily develop profound thoughts about the movies and series they watch, or perhaps they do but it may take some time before initial impressions transform into well-articulated reflections [see 51 for a similar finding for readers’ reflections on written stories]. Future research could examine this possibility by measuring reflection at multiple points in time. Research in this direction could furthermore examine alternative movie characteristics that may influence viewers’ reflection. For example, close-ups have been found to affect viewers’ spontaneous attribution of mental states to characters [52]. Thinking about what characters think and feel could be a precursor of reflective thoughts.

### Limitations

The movies used in this study were relatively short, ranging from 4 to 11 minutes. Although this is similar to the length of stimuli used in previous studies on tragic films and reflection [8–10], the results of this study may not reflect what happens when people watch full-length movies. A second limitation is that the role of the character who died differed between and within the conditions: it was the main character’s (ex-)boyfriend in the movies with death as a resolution (*Song of the Rain* and *Message from Tar Creek*), and it was a friend or it were parents in the movies with death as a triggering event (*Street Flame* and *Lost in the Smoke*, respectively). The type of death also varied: characters died in a war (*Song of the Rain*), committed suicide (*Message from Tar Creek*), had a fatal accident (*Street Flame*) or died in a fire (*Lost in the Smoke*). Although the four movies were equally absorbing and moving and elicited equal levels of viewers’ identification, these differences are potential confounds.

At the same time, the use of existing short movies is a strength of this study as it increases the ecological validity, and, while it is hard to assess the representativeness of the stimulus movies in an objective way, we used multiple movies within one condition to increases the study’s generalizability. Nonetheless, studies in which people watch full movies, with similar deaths, would be valuable to further advance our understanding of the effects of eudaimonic entertainment. Studies in this direction could furthermore use alternative methods, such as interviews or focus groups, to gain in-depth insights into viewers’ experiences of death in eudaimonic movies and to ultimately arrive at a more thorough understanding of how these experiences might influence viewers’ attitudes towards life and death.

## Supporting information

S1 Movie Stimulus movies. (DOCX)
