# Supplementary material for: Do tragic movies about death make you think? Exploring effects of the role of death in the narrative structure of eudaimonic movies on viewers’ reflection, death attitudes, and posttraumatic growth
Source: PLoS One. 2025 May 19;20(5):e0323739. doi: 10.1371/journal.pone.0323739 (PMC12088012; doi:10.1371/journal.pone.0323739)
Supplement: S1 Movie — (DOCX) [file pone.0323739.s001.docx]

**Stimulus movies**

Chen, L, producer; Liang, director. The Song of the Rain [Film], 2017. <https://www.youtube.com/watch?v=kXnmphnF7v8>

O’Brien, N, director; Gallagher, M, Olson, W, producers. A Message from Tar Creek [Film], 2016. <https://www.youtube.com/watch?v=8YMNMk39N5E>

Orgill, B., director and producer. Lost in the Smoke [Film], 2019. <https://vimeo.com/335920944>

Propper, K, director; Alvarado, J, producer. Street Flame [Film], 2019. <https://www.youtube.com/watch?app=desktop&v=xTlUa-l8zBw>
